# Associations between physical activity and dietary live microbe intake with non-alcoholic fatty liver disease: findings from the NHANES, 2007–2018

**DOI:** 10.7189/jogh.16.04119

**Published:** 2026-08-14

**Authors:** Shu Ding, Xiaoke Hu, Jia Liu

**Affiliations:** 1 Department of Nursing, Beijing Chao-Yang Hospital, Capital Medical University, Beijing, China; 2 Department of Endocrinology, Beijing Chao-Yang Hospital, Capital Medical University, Beijing, China

**Keywords:** dietary live microbe intake, non-alcoholic fatty liver disease, physical activity, triglyceride-glucose index

## Abstract

**Background:**

Physical activity (PA) and dietary live microbe intake protect against non-alcoholic fatty liver disease (NAFLD) by improving insulin resistance, yet whether they act through shared metabolic pathways – such as those reflected by the triglyceride-glucose (TyG) index – remains unclear. We aimed to investigate the independent and combined associations of PA and dietary live microbe intake with NAFLD and to explore the mediating effect of the TyG index.

**Methods:**

We retrieved data from the National Health and Nutrition Examination Survey 2007–2018, which defined NAFLD by the US fatty liver index, PA using the self-reported Global Physical Activity Questionnaire, and dietary live microbe intake levels through 24-hour dietary recall data. We assessed individual and joint effects of PA and dietary live microbe intake *via* weighted multivariate logistic regression models and explored the mediating effect of the TyG index *via* causal mediation analysis.

**Results:**

Our analysis encompassed 10 387 participants. The multivariate-adjusted odds ratios (ORs) were 0.84 (95% confidence interval (CI) = 0.72–0.99; *P* = 0.040) for NAFLD patients with sufficiently high PA and 0.78 (95% CI = 0.66–0.92; *P* = 0.003) for those with relatively high dietary live microbe intake. In the joint analysis, individuals with sufficiently high PA and higher dietary live microbe intake had the lowest risk of NAFLD (OR = 0.68; 95% CI = 0.56–0.84, *P* < 0.001), particularly those aged <65 years. The TyG index mediated 40.80% of the association between PA and NAFLD, as well as 28.44% of the association between dietary live microbe intake and NAFLD.

**Conclusions:**

Our findings suggest that public health strategies promoting both regular physical activity and consumption of foods rich in live microbes may offer a synergistic approach for NAFLD prevention, particularly in adults under 65 years of age.

Non-alcoholic fatty liver disease (NAFLD) has emerged as a leading cause of chronic liver disease globally [1]. Its estimated global prevalence, which is currently at 30.0%, has previously increased from 25.26% in 1990–2006 to 38.2% in 2016–2019 [2]. The condition is primarily driven by insulin resistance, which leads to increased free fatty acid and lipid accumulation in the liver [3]. Inflammation and oxidative stress further contribute to the progression from simple steatosis to non-alcoholic steatohepatitis [4], while both the gut microbiota and genetic factors have the same effect on development of NAFLD [5].

The management of NAFLD primarily focuses on lifestyle changes aimed at achieving weight loss, such as increasing exercise and maintaining a balanced diet. It has been demonstrated, for example, that regular physical activity (PA) can improve peripheral sensitivity to insulin, as well as reduce adipocyte lipolysis and the delivery of free fatty acids to the liver, thereby reducing the hepatic fat content [6]. While increased PA is beneficial for NAFLD patients to some extent, its effects could be further strengthened through a balanced diet. In this context, the consumption of dietary live microbes has gained considerable attention due to their potentially favourable effects on human health [7]. The consumption of fermented foods that contain such microbes, such as yogurt, has also been shown to improve metabolic and immune homeostasis [8–10]. Sanders et al [11] assessed the number of live microbes that consumed in the diet and categorised all foods into low (<10^4^ CFU/g), medium (10^4^–10^7^ CFU/g), or high (>10^7^ CFU/g) levels of live microbes. Multiple studies have revealed that higher dietary live microbe intake is associated with a reduced risk of metabolic diseases such as obesity, NAFLD, cardiovascular diseases (CVDs), osteoporosis, and diabetic kidney disease [12–16]. Yet the evidence regarding the joint effect of PA and dietary live microbe intake on the risk of NAFLD remains limited.

A key mechanisms in the development of NAFLD is insulin resistance [17]. Studies have demonstrated that adequate PA or exercise is effective in improving insulin sensitivity [18,19], and that dietary live microbe intake could be associated with lower insulin resistance [20]. The triglyceride-glucose (TyG) index, a recently identified alternative biomarker of insulin resistance, has garnered interest because of its straightforward calculation, which relies on fasting triglycerides and glucose, both of which are easily accessible [21]. To the best of our knowledge, no studies have examined whether PA and dietary live microbe intake influence NAFLD through the TyG index. Therefore, we employed the National Health and Nutrition Examination Survey (NHANES) data and Sanders and colleagues’ assessment method for dietary live microbe intake levels [11] to investigate the associations of PA and dietary live microbe intake with NAFLD, as well as the mediating role of TyG.

## METHODS

### Study design and participants

The NHANES uses stratified, multistage probability sampling method to systematically collect nationally representative health-related data on the non-institutionalised population (children and adults) in the USA [22]. It has been carried out every two years since 1990. The National Center for Health Statistics’ Research Ethics Review Board approved the study, with all participants providing informed consent.

We retrieved data from six consecutive cycles of the survey: 2007–2008, 2009–2010, 2011–2012, 2013–2014, 2015–2016, and 2017–2018. Within the NHANES, NAFLD was defined by the use of the United States fatty liver index (USFLI), which is calculated based on age, race, waist circumference, gamma-glutamyl transferase (GGT), fasting insulin, and fasting glucose.

We excluded pregnant individuals and those aged <20 years, as the definition and clinical relevance of NAFLD, lifestyle exposures (*e.g.* physical activity and dietary intake), and related metabolic covariates differ substantially between adolescents and adults. We further excluded participants with missing data on GGT, race, age, or waist circumference, and those with other potential causes of fatty liver: excessive alcohol consumption (more than three drinks per day for men and more than two drinks per day for women); infection with hepatitis B (positive for hepatitis B surface antigen or core antibody) or hepatitis C (positive for hepatitis C antibody or hepatitis C virus RNA); and steatogenic medications use (amiodarone, corticosteroids, methotrexate, valproate, and tamoxifen) for more than 90 days. Lastly, we excluded participants with incomplete information about their diet, PA, education level, or body mass index (BMI).

We limited our analysis to cycles up to 2017–2018 for two reasons. First, data collection for the NHANES 2019–2020 was severely disrupted by the COVID-19 pandemic, leading to differences in protocols and potential biases. Second, data from the 2021–2022 cycle were only partially released and several key components we required for our analysis were unavailable. Using the six consecutive cycles from 2007–2018 ensured data consistency, comparable measurement protocols, and a sufficiently large sample size for robust analysis.

### Definition of NAFLD

As mentioned above, the NHANES defines NAFLD using the USFLI score, with a score exceeding 30 indicating the presence of NAFLD after excluding other causes of hepatic steatosis. The USFLI is a validated, non-invasive tool that has demonstrated satisfactory diagnostic performance for NAFLD in large epidemiological surveys like NHANES where liver imaging data are not routinely available, with an area under the receiver operating characteristic curve of 0.80 (sensitivity of 62%, specificity of 88%) when validated against imaging-based assessments [23]. The USFLI ranges from 0 to 100 and was calculated *via* the following formula, where ‘non-Hispanic black’ and ‘Mexican American’ had values of 1 if the participant was of that ethnicity and 0 if not of that ethnicity [23]:

*y* = 0.3458 × *Mexican American* − 0.8073 × *Non-Hispanic Black* + 0.0093 × *age* + 0.6151 × *ln*(*GGT*) + 0.0249 × *waist circumference* + 1.1792 × *ln*(*insulin*) + 0.8242 × *ln*(*glucose*) − 14.7812

*USFLI* = *e^y^*/(1 + *e^y^*) × 100

### Measurement of PA

In the NHANES, PA was self-reported by participants using the Global Physical Activity Questionnaire (GPAQ). In accordance with the GPAQ analysis guidelines, the PA of each participant was converted to metabolic equivalent (MET) minutes of moderate-to-vigorous PA per week. In accordance with the GPAQ analysis guide [22], the MET values assigned in the NHANES were 8.0 for vigorous work-related and vigorous leisure-time activities, and 4.0 for moderate work-related activities, walking or bicycling for transportation, and moderate leisure-time activities.

The scores for each type of PA were calculated via the formula MET × weekly frequency × duration of each PA, which was added together to obtain the total PA score for each participant. According to the American Physical Activity guidelines [24], adults should perform at least 150 minutes of moderate-intensity PA or 75 minutes of vigorous-intensity PA per week (equivalent to 600 MET-minutes per week). Here, we separate PA into two levels: insufficiently active (<600 MET minutes/week) and sufficiently active (≥600 MET minutes/week).

### Measurement of dietary live microbe intake

Dietary live microbial intake in the NHANES was estimated *via* 24-hour dietary recall data from day 1 and day 2. Sanders *et al*. [11] developed a method to calculate the number of live microbes (per gram) in 9388 food codes spread across 48 subgroups in the NHANES database. Foods were classified into three categories based on their estimated live microbial content: low (<104 CFU/g), medium (104–107 CFU/g), and high (>107 CFU/g). A fourth category, medium-high (MedHi), was also created to aggregate consumers of foods from the medium, high, or both medium and high categories [11]. Generally, foods pasteurised or processed at high temperatures are grouped into the low level category, unpeeled fresh vegetables and fruits into the medium level category, and unpasteurised fermented foods and probiotic supplements in the high level category.

We aggregated the amounts of medium- and high-level foods consumed per person on day 1 and day 2 (in 100 g) and defined the average as MedHi, which we then converted into a categorical variable for analysis. This created two groups: people who consumed less (G1) *vs*. more (G2) MedHi foods than the median MedHi intake.

### Other covariates

In the NHANES, hypertension was defined as systolic blood pressure ≥140 mm Hg and/or diastolic blood pressure ≥90 mm Hg, self-reported hypertension, or self-reported use of antihypertensive medication. The participants were considered to have diabetes mellitus if their fasting blood glucose level was ≥126 mg/dl, if their two-hour glucose level was ≥200 mg/dl after an oral glucose tolerance test, if their glycosylated haemoglobin A1c (HbA1c) level was ≥6.5%, if their self-reported diagnosis by doctors, or if they used insulin or oral hypoglycaemic medication. Hyperlipidaemia was defined as a self-reported high cholesterol level, self-reported use of cholesterol-lowering medications, or laboratory examination indicating TC≥200 mg/dl, triglycerides ≥150 mg/dl, LDL-C≥130 mg/dl, and HDL<40 mg/dl (male) or <50 mg/dl (female). CVD was defined on the basis of a self-reported history of heart failure, coronary artery disease, angina, heart attack, or stroke. The TyG index was calculated as *ln* (*fasting triglycerides* (*mg/dl*) × *glucose* (*mg*/*dl*)/2). In the NHANES, energy intake, protein intake, carbohydrate intake, total fat intake, and fibre intake were calculated via the average of data from two 24-hour dietary recalls. The total Health Eating Index 2020 score ranges from 0 to 100, with a higher score indicating greater overall dietary quality (DQ). Participants with a two-day HEI-2020 average score of more than 60 were considered to have adhered to the dietary guidelines [24,25]. Therefore, we classified DQs into two levels: unqualified (score <60) and qualified (score ≥60).

Other potential covariates included age, sex, race (Mexican, other Hispanic, non-Hispanic White, non-Hispanic Black, other races), education level (below 9th grade, 9–11th grade, high school graduate/general education development or equivalent, some college or associate of arts degree, college graduate or above), marital status (married, never married, other), household income-poverty ratio (PIR: <1.3, 1.3–3.5, >3.5), and smoking status (never, former, or current). BMI, which was calculated as weight divided by height squared, was classified as normal (<25 kg/m^2^), overweight (25–30 kg/m^2^), or obese (≥30 kg/m^2^).

### Statistical analyses

We used sampling weights for all analyses, in accordance with the NHANES analytical [22]. Specifically, we calculated twelve-year weights for the 2007–2018 estimates by dividing the two-year weights by six. Given the limitations of normality tests in large samples, we based our choice of statistical methods for continuous variables on their empirical distribution, scale of measurement, and conventions in epidemiological analysis. We presented categorical variables as frequencies and percentages, and compared them using the Rao-Scott χ^2^ test. For continuous variables, we summarised those with approximately symmetric distributions as means and standard deviations, comparing them using Student’s *t-*tests, and those with skewed distributions as medians and interquartile ranges (IQRs), comparing them using the Kruskal-Wallis test.

We constructed a logistic regression model to assess the associations of PA and dietary live microbe intake levels with NAFLD among participants. Model 1 was adjusted for age, sex, race, BMI, hypertension, diabetes, hyperlipidaemia, and CVD, while model 2 was further adjusted for energy intake, protein intake, carbohydrate intake, total fat intake, fibre intake, and DQ. We performed subgroup analyses by age (<65 years and ≥65 years). Lastly, we explored the mediation effect of TyG via causal mediation analysis.

We performed all statistical analyses in R, version 4.4.1 (R Foundation for Statistical Computing, Vienna, Austria). A two-tailed *P* value of <0.05 was considered statistically significant.

## RESULTS

We retrieved data from six consecutive cycles of the survey: 2007–2008, 2009–2010, 2011–2012, 2013–2014, 2015–2016, and 2017–2018. Within the NHANES, NAFLD was defined by the use of the United States fatty liver index (USFLI), which is calculated based on age, race, waist circumference, GGT, fasting insulin, and fasting glucose. After all exclusions, 10 387 participants were included in the final analysis (Figure S1 in the **Online Supplementary Document**).

### Baseline characteristics

Our analysis included 10 387 American adults (weighted population: 159 770 878) (**Table 1**). The weighted median age was 49 years (IQR = 35–62), and 46.87% were male. There were 3685 participants with and 6702 without NAFLD. Compared to participants without NAFLD, those with NAFLD were older; had a higher proportion of males; had a higher proportion of Mexican American or non-Hispanic White populations; were less educated; had a higher proportion of married individuals; had lower PIRs; had higher BMIs; had a higher proportion of individuals with hypertension, diabetes, hyperlipidaemia, and CVD; and had higher energy, protein, and total fat intake and lower fibre intake. They also engaged in less PA, had lower dietary live microbe intake, and a higher TyG index than those without NAFLD.

**Table 1:** Baseline characteristics of the study population*

	Overall	Non-NAFLD	NAFLD	*P*-value
**Unweighted number**	10 387	6702	3685	
**Weighted number**	159 770 878	106 653 748	53 117 129	
**Age in years, MD (IQR)**	49 (35–62)	46 (32–60)	54 (42–65)	<0.001
**Gender**				<0.001
Male	4824 (46.87)	2896 (43.19)	1928 (54.26)	
Female	5563 (53.13)	3806 (56.81)	1757 (45.74)	
**Race**				<0.001
Mexican American	1524 (7.64)	693 (5.65)	831 (11.64)	
Other Hispanic	1117 (5.63)	689 (5.58)	428 (5.72)	
Non-Hispanic White	4537 (68.75)	2863 (67.62)	1674 (71.02)	
Non-Hispanic Black	2009 (10.50)	1582 (12.86)	427 (5.77)	
Other	1200 (7.48)	875 (8.29)	325 (5.85)	
**Education level**				<0.001
Below 9th grade	1098 (5.40)	539 (4.26)	559 (7.69)	
9–11th grade	1369 (9.46)	793 (8.14)	576 (12.13)	
High school graduate/GED or equivalent	2211 (21.59)	1414 (20.98)	797 (22.81)	
Some college or AA degree	2993 (29.90)	1945 (28.73)	1048 (32.23)	
College graduate or above	2716 (33.65)	2011 (37.89)	705 (25.14)	
**Marital status**				<0.001
Married	5710 (59.59)	3553 (58.05)	2157 (62.67)	
Never married	1658 (15.68)	1237 (17.62)	421 (11.80)	
Other	3019 (24.73)	1912 (24.33)	1107 (25.53)	
**PIR**				0.011
<1.3	2788 (18.00)	1689 (17.28)	1099 (19.42)	
1.3–3.5	4542 (39.95)	2913 (39.32)	1629 (41.22)	
>3.5	3057 (42.05)	2100 (43.40)	957 (39.35)	
**BMI in kg/m2**				<0.001
<25	3015 (29.70)	2829 (42.73)	186 (3.54)	
25–30	3419 (32.69)	2443 (37.30)	976 (23.43)	
≥30	3953 (37.61)	1430 (19.97)	2523 (73.03)	
**Smoking status**				0.065
Never	1324 (11.64)	919 (12.34)	405 (10.23)	
Ever	301 (2.48)	193 (2.56)	108 (2.34)	
Now	8762 (85.88)	5590 (85.10)	3172 (87.43)	
**Hypertension**				<0.001
No	5853 (61.39)	4341 (70.38)	1512 (43.33)	
Yes	4534 (38.61)	2361 (29.62)	2173 (56.67)	
**Diabetes**				<0.001
No	8037 (83.32)	5875 (91.91)	2162 (66.06)	
Yes	2350 (16.68)	827 (8.09)	1523 (33.94)	
**Hyperlipidaemia**				<0.001
No	2651 (26.58)	2210 (34.04)	441 (11.60)	
Yes	7736 (73.42)	4492 (65.96)	3244 (88.40)	
**CVD**				<0.001
No	9163 (90.75)	6123 (93.51)	3040 (85.21)	
Yes	1224 (9.25)	579 (6.49)	645 (14.79)	
**PA**				<0.001
Insufficiently high	4226 (35.98)	2474 (32.44)	1752 (43.09)	
Sufficiently high	6161 (64.02)	4228 (67.56)	1933 (56.91)	
**MedHi**				<0.001
G1	5193 (45.21)	3240 (43.40)	1953 (48.83)	
G2	5194 (54.79)	3462 (56.60)	1732 (51.17)	
**Energy intake in kcal/d, MD (IQR)**	1961 (1515.50–2506.00)	1 938 (1508.50–2471.00)	2008 (1522.50–2587.00)	0.006
**Protein intake in g/d, MD (IQR)**	77 (58–99)	76 (57–97)	79 (59–103)	<0.001
**Carbohydrate intake in g/d, MD (IQR)**	232.69 (175.79–303.21)	232.83 (175.10–302.23)	232.52 (176.83–307.58)	0.200
**Total fat intake in g/d, MD (IQR)**	74.60 (54.87–101.48)	72.66 (54.11–98.77)	79.29 (56.86–105.74)	<0.001
**Fibre intake in g/d, MD (IQR)**	15.75 (11.15–21.85)	15.95 (11.20–22.10)	15.30 (11.00–21.30)	0.024
**DQ, HEI-2020**				<0.001
Unqualified	6485 (74.75)	4022 (71.56)	2463 (81.14)	
Qualified	2335 (25.25)	1674 (28.44)	661 (18.86)	
**TyG index, MD (IQR)**	8.53 (8.11–8.98)	8.32 (7.97–8.69)	8.98 (8.61–9.37)	<0.001

AA – associate of arts, BMI – body mass index, CVD – cardiovascular disease, DQ – dietary quality, GED – general education development, G1 – intake of MedHi food below the median level, G2 – intake of MedHi food above the median level, HEI-2020 – Health Eating Index 2020, IQR – interquartile range, MD – median, MedHi – medium- and high-level live microbial foods, MET – metabolic equivalent minutes, NAFLD – non-alcoholic fatty liver disease, PA – physical activity, PIR – ratio of family income to poverty

*Values are presented as n (%) unless specified otherwise. Weighted number represents the estimated population size after applying NHANES survey weights.

**Figure 1:**
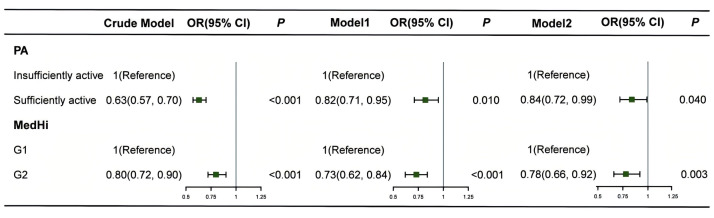
Association of PA and dietary live microbe intake levels with NAFLD among participants. Model 1 was adjusted for age, gender, race, BMI, hypertension, diabetes, hyperlipidaemia and cardiovascular diseases. Model 2 was further adjusted for energy intake, protein intake, carbohydrate intake, total fat intake, fibre intake, and DQ.

In unweighted and weighted univariate logistic regression analyses, participants with sufficiently high PA had a reduced risk of NAFLD compared to those with insufficiently low PA (OR = 0.63; 95% CI = 0.57–0.70, *P* < 0.001). Individuals who consumed MedHi food above the median level had a lower prevalence of NAFLD than did those with intakes of MedHi food below the median level (OR = 0.80; 95% CI = 0.72–0.90, *P* < 0.001). In both model 1 and 2, participants with sufficient PA and higher levels of MedHi food intake had a reduced risk of NAFLD (**Figure 1**).

**Figure 2:**
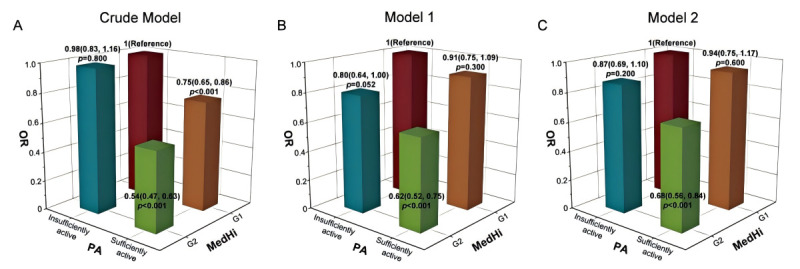
Joint association of PA and dietary live microbe intake levels with NAFLD. Model 1 was adjusted for age, gender, race, BMI, hypertension, diabetes, hyperlipidaemia, and cardiovascular diseases. Model 2 was further adjusted for energy intake, protein intake, carbohydrate intake, total fat intake, fibre intake, and DQ.

The combination of adequate PA and increased dietary live microbe intake was associated with a decreased risk of NAFLD (**Figure 2**). Specifically, compared with the groups with combined insufficient PA (<600 MET-min/week) and MedHi food intake below the median level (G1), the ORs for NAFLD in the groups with sufficiently high PA (≥600 MET-min/week) and MedHi food intake above the median level (G2) were 0.54 (95% CI = 0.47–0.63) for the crude model (*P* < 0.001); 0.62 (95% CI = 0.52–0.75) for model 1 (*P* < 0.001), and 0.68 (95% CI = 0.56–0.84) for model 2 (*P* < 0.001).

**Figure 3:**
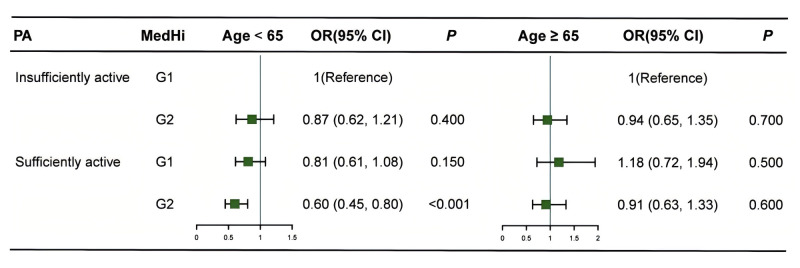
Joint association of PA and dietary live microbe intake levels with NAFLD stratified by age. Model 1 was adjusted for age, gender, race, BMI, hypertension, diabetes, hyperlipidaemia, and cardiovascular diseases. Model 2 was further adjusted for energy intake, protein intake, carbohydrate intake, total fat intake, fibre intake, and DQ.

Our stratified analysis showed that sufficient PA and higher levels of MedHi food intake were associated with a lower risk of NAFLD among participants aged <65 years (OR = 0.60; 95% CI = 0.45–0.80, *P* < 0.001) (**Figure 3**). Our mediation analysis showed that TyG mediated 40.80% of the association between PA and NAFLD, as well as 28.44% of the association between dietary live microbe intake and NAFLD (Figure S2 in the **Online Supplementary Document**).

## DISCUSSION

We found that sufficient PA and increased dietary live microbe intake were independently associated with a reduced risk of NAFLD. Furthermore, a higher level of dietary live microbe intake significantly enhanced the protective effect of PA against NAFLD, especially among individuals aged <65 years. These associations remained significant even after adjusting for multiple confounding factors, including gender, race, BMI, hypertension, diabetes, hyperlipidaemia, CVD, energy intake, protein intake, carbohydrate intake, total fat intake, fibre intake, and DQ. Moreover, TyG mediated the associations of PA and dietary live microbe intake with NAFLD.

An increasing number of epidemiological studies have shown that sedentary behaviour and insufficient PA are associated with increased risks of obesity, diabetes, NAFLD, metabolic syndrome, and cardiovascular diseases [24–26]. In contrast, higher levels of moderate-to-vigorous PA were associated with lower risks of adverse liver outcomes, including steatosis, fibrosis, and cirrhosis [27]. A previous study showed that even short-term exercise (60 minutes per day for seven days) improved hepatic markers of apoptosis in obese patients with NAFLD [28]. Furthermore, a prospective study showed that eight weeks of aerobic and resistance exercise improved the expression of markers of hepatic steatosis, inflammation, and fibrosis independent of weight loss in patients with NAFLD [29]. Both the previously reported studies and our analysis indicated that sufficient PA had significant benefits on NAFLD, even in the absence of weight loss. Consistent with these findings, we observed the beneficial effect of sufficiently high PA (≥600 MET-min/week) in reducing the risk of NAFLD.

In recent years, changes in dietary patterns have contributed to an increasing incidence of NAFLD. Diet serves not only as a vital source of energy and nutrients, but also as the primary pathway for the intake of live microbes [6,11,13]. The Food and Agriculture Organization of the United Nations and the World Health Organization define probiotics as live microbes that, when administered in adequate amounts, prove beneficial for the consumer’s health [30]. These live microbes are found not just in fermented foods such as yogurt, but also in other foods such as fresh, unpeeled fruits and vegetables [31]. Our findings suggested that individuals who consumed relatively high levels of live microbes had a reduced risk of NAFLD, which is consistent with previous findings [13]. These symbiotic or harmless dietary live microbes that reach the intestine may interact with the resident gut microbiota to regulate lipid metabolism and glucose metabolism [32,33], inhibit oxidative stress and chronic low-grade inflammation [34,35], and improve the gut microbiota composition [36], thereby preventing and ameliorating obesity and obesity-related chronic diseases. While we examined total intake of medium/high microbial-content foods, future research could investigate associations with specific food categories (*e.g.* fermented dairy vs unpeeled produce). Our preliminary exploratory analysis suggested that both fermented foods and produce contributed to the overall inverse association, indicating that the benefits may not be driven by a single food type. Furthermore, exploratory subgroup analyses suggested that the inverse associations of PA and dietary live microbes with NAFLD might be more pronounced in individuals without diabetes, but were consistently observed across different body weight categories. Future larger studies are needed to confirm potential effect modification by metabolic comorbidities.

We noted that an increased dietary live microbe intake significantly enhanced the protective effect of PA against NAFLD. Previous research similarly suggests that combining exercise with dietary interventions results in greater improvements in alanine aminotransferase levels compared to engaging in exercise alone among patients with NAFLD [37]. These findings also suggest that PA and dietary live microbe intake may influence NAFLD through shared mechanisms.

Some studies have shown that adequate PA or exercise can significantly enhance insulin sensitivity [18,19], while a previous study indicated a negative connection between dietary live microbe intake and the homeostatic model assessment of insulin resistance [20]. To further investigate this relationship, we performed mediation analysis focused on TyG, which serves as an indicator of insulin resistance. We found that TyG mediated the associations of PA and dietary live microbe intake with NAFLD, suggesting that PA and dietary live microbe intake may influence NAFLD through insulin sensitivity. Although the specific mechanisms remain unclear, we speculate that the gut microbiota may play a key role in this association. Previous studies have shown that various risk factors, such as obesity, physical inactivity, and high-fat, high-sugar diets, can alter the composition of the gut microbiome, leading to inflammation, insulin resistance, and abnormal lipid metabolism, which aggravate the development of NAFLD [38]. Additionally, butyrate derived from probiotics can stimulate the secretion of the gut hormone glucagon-like peptide-1, promoting insulin release [39]. These findings suggest that a combined intervention of PA and dietary live microbe is likely to more comprehensively improve NAFLD. Notably, we found that a higher dietary live microbe intake enhanced the effect of PA in reducing NAFLD risk, particularly among individuals aged <65 years. This may be attributed to the influence of age on both the quantity and diversity of gut microbiota [40]. Therefore, personalising dietary live microbe intake and PA levels for different age groups would be more targeted and effective.

To our knowledge, this was the first large-scale epidemiological study to explore the joint association of PA and dietary live microbe intake with NAFLD. However, it has some limitations. First, its cross-sectional design does not allow us to draw conclusions on the causal relationship of PA and dietary live microbe intake with NAFLD. A large prospective cohort study should be conducted in the future to explore such a provide more robust evidence on this association. Second, our results are subject to recall bias, as PA and dietary live microbe intake levels were based on self-reported data in the NHANES and may be subject to non-differential misclassification, which might have biased the observed associations toward the null. Therefore, our estimates of the protective effect of PA on NAFLD may be conservative. Third, the classification of dietary live microbe intake relied on expert-estimated microbial loads per food group rather than empirical quantification for individual food items [11]. This approach cannot account for variations due to brand, processing, or storage, and does not provide an absolute count of live microbes consumed. Fourth, the NHANES defined NAFLD using the USFLI, a validated surrogate marker. While practical for large-scale studies, USFLI cannot differentiate simple steatosis from non-alcoholic steatohepatitis or assess fibrosis stage and hepatic inflammation. Future studies incorporating liver biopsy or advanced imaging are warranted to confirm these associations across the spectrum of NAFLD severity. Fifth, despite adjusting for numerous covariates, residual confounding remains possible. Dietary live microbe intake may be correlated with health-conscious behaviours (*e.g.* overall dietary patterns, health care utilisation, or other positive lifestyle choices not fully captured by our variables) which were not measured in the NHANES. This ‘healthy user’ bias could partly explain the observed associations. Finally, this study focused on adults from the USA only, so our findings still need to be validated in other populations.

## CONCLUSIONS

We found that individuals with adequate PA and higher dietary live microbe intake had a reduced risk of NAFLD, both independently and in combination, especially in adults aged <65 years. Furthermore, TyG mediated the relationships of PA and dietary live microbe intake with NAFLD. These findings underscore the importance of PA and dietary live microbe intake in improving NAFLD, which may consequently have significant public health implications.

## Additional material


Online Supplementary Document
