# Supplementary material for: Associations between physical activity and dietary live microbe intake with non-alcoholic fatty liver disease: findings from the NHANES, 2007–2018
Source: J Glob Health. 2026 Aug 14;16:04119. doi: 10.7189/jogh.16.04119 (PMC13474058; doi:10.7189/jogh.16.04119)
Supplement: Supplementary file 1 [file jogh-16-04119-s001.pdf]

Supplement to: Ding S, Hu X, Liu J. Associations between physical activity and dietary live microbe intake with non-alcoholic fatty liver disease: findings from the NHANES, 2007–2018. J Glob Health. 2026;16:04119.

Appendix. Outline of JoGH's Guidelines for Reporting Analyses of Big Data Repositories Open to the Public (GRABDROP) items

JoGH guideline item	Purpose	Response
1. Please list all papers published by each co-author in previous three years that were based on secondary analysis of a big data repository	Preventing publications from possible 'paper mills' and authorships granted without genuine contribution	Integrative Multi-Omics Profiling of Dynamic Body Mass Index–Systolic Blood Pressure Trajectories in Obesity for Precision Risk Stratification of Heart Failure Subtypes [2026, Jia Liu, DOI: 10.3390/biomedicines14071473]
2. Please explain the key elements of your study design and the use of the available datasets that make your study an original scientific contribution	Preventing duplicate publication where published information is reorganised and republished	Most existing studies only separately explored the correlation between single lifestyle factor (physical activity or fermented food/live microbe intake) and NAFLD using NHANES data. This study innovatively combined two exposure factors: physical activity and dietary live microbe intake, analysed their independent and synergistic joint correlation with NAFLD. We further conducted age-stratified subgroup analysis and causal mediation analysis to verify the mediating role of the TyG index (insulin resistance biomarker) between the two exposures and NAFLD. This multi-angle analytical framework based on 6 cycles of nationally representative NHANES 2007–

		2018 data forms the original scientific contribution of this paper.
3. Please list all publications that addressed similar research questions in the same dataset and indicate where you cited them in your paper	Preventing duplicate publication where other groups have already conducted similar research in the same dataset	Multiple prior studies used NHANES data to analyse single lifestyle and NAFLD risk, including Reference 6, Reference 13, Reference 20. All relevant literatures are fully cited in the Introduction and Discussion sections of the manuscript.
4. Please explain how you addressed multiple testing through an appropriately rigorous statistical threshold and indicate this in the methods section	Preventing publication of false positive associations which are of low value unless a rigorous statistical method accounted for multiple testing	We adopted a two-tailed $P < 0.05$ as unified statistical significance threshold for all regression, subgroup and mediation analyses. Multiple confounding factors including demographics, metabolic diseases and total dietary intake were stepwise adjusted in two regression models to reduce false positive bias from multiple variable screening. The complete statistical processing rules for controlling multiple testing are fully described in the Methods section of the manuscript.
5. Please declare to what extent have AI chatbots been used in developing your paper and to which parts of the paper did they contribute	Preventing the improper use of AI chatbots in developing the key elements of the paper and ensuring reproducibility and transparency	AI tools were only applied for minor English grammar correction and sentence polishing of the manuscript. No AI tools participated in study design, data cleaning, result interpretation, core scientific discussion or drawing conclusions.

AI – artificial intelligence, JoGH – Journal of Global Health
